# A Hybrid SAR/ISAR Approach for Refocusing Maritime Moving Targets with the GF-3 SAR Satellite

**DOI:** 10.3390/s20072037

**Published:** 2020-04-04

**Authors:** Zhishuo Yan, Yi Zhang, Heng Zhang

**Affiliations:** 1 Department of Space Microwave Remote Sens. Systems, Aerospace Information Research Institute, Chinese Academy of Sciences, Beijing 100190, China; yanzhishuo17@mails.ucas.ac.cn (Z.Y.); zhangyi@mail.ie.ac.cn (Y.Z.); 2 School of Electronics, Electrical and Communication Engineering, University of Chinese Academy of Sciences, Beijing 100049, China

**Keywords:** synthetic aperture radar (SAR), moving targets, inverse SAR (ISAR), motion compensation, hybrid SAR/ISAR, improved rank-one phase estimation (IROPE), Gaofen-3 (GF-3)

## Abstract

Due to self-motion and sea waves, moving ships are typically defocused in synthetic aperture radar (SAR) images. To focus non-cooperative targets, the inverse SAR (ISAR) technique is commonly used with motion compensation. The hybrid SAR/ISAR approach allows a long coherent processing interval (CPI), in which SAR targets are processed with ISAR processing, and exploits the advantages of both SAR and ISAR to generate well-focused images of moving targets. In this paper, based on hybrid SAR/ISAR processing, we propose an improved rank-one phase estimation method (IROPE). By using an iterative two-step convergence approach in the IROPE, the proposed method achieves accurate phase error, maintains robustness to noise and performs well in estimating various phase errors. The performance of the proposed method is analyzed by comparing it with other focusing algorithms in terms of processing simulated data and real complex image data acquired by Gaofen-3 (GF-3) in spotlight mode. The results demonstrate the effectiveness of the proposed method.

## 1. Introduction

Synthetic aperture radar (SAR) provides all-weather, day–night, wide-range high-resolution imaging capabilities for a wide range of applications in Earth science and climate change research, marine detection and imaging, and disaster monitoring [1]. Nevertheless, SAR uses the motion of the radar, ignoring target motion, to coherently synthesize a large aperture that provides a narrow synthesized beam, and it thus has a high resolution across the range. Therefore, in the ship detection and classification scenario, ships are always defocused with conventional SAR processing because of individual motion and sea waves. Inverse SAR (ISAR) uses the rotational motion of the targets, ignoring radar motion, to distinguish different relative velocities through coherent processing and Doppler effects and to form the synthetic aperture. Thus, ISAR processing is more adaptable to the moving scenario since it is superior in imaging moving targets undergoing complex unknown motions [2]. However, due to the unpredictability of non-cooperative targets, motion compensation in ISAR imaging is a challenging task and usually includes two steps: range tracking and Doppler tracking, i.e., coarse phase compensation and fine phase compensation [3]. This paper focuses on the study of fine phase compensation, which is more sensitive than range tracking. Moreover, during the operation of SAR for a moving target, both the radar and target are in motion, which means that the processing of the moving target must combine the processes of SAR (radar motion) and ISAR (target motion) [4,5]. Hybrid SAR/ISAR [6,7] processing is such an approach to optimally process SAR data by treating target and radar platform motions on an equal footing, which takes advantage of ISAR processing to generate the focused image of the moving target in SAR.

Ideally, the phase of the echo signal in the range and azimuth profiles varies linearly during the processing time. However, due to various factors, there exist undesired phase changes in the echo signal, which are collectively referred to as phase error [8]. Phase error, which is divided into low-frequency, high-frequency, and random phase errors, causes geometric distortion, resolution degradation, false targets, and reduced signal-to-noise ratio (SNR), thus resulting in poor image quality. Low-frequency errors encompass linear phase errors, quadratic phase errors (QPEs), and so on. The low-frequency phase errors primarily affect the main lobe of the system impulse response, while high-frequency errors affect the sidelobe regions. Random phase errors cause multiple pairs of echoes around the target, and the main lobe energy is reduced [9].

To estimate the phase error and refocus the images, many fine phase compensation algorithms have been proposed, which are roughly divided into parametric and nonparametric algorithms. The parametric algorithms include the Mapdrift (MD) method [10,11], the phase difference (PD) method [12] and methods of parameter estimation [13]. MD and PD methods are easy to implement, but they only compensate for QPEs, which limits their applications. Furthermore, Chen et al. [14] proposed a parametric sparse representation method. The acceleration and third-order phase were considered in [15,16]. Tang et al. [17] achieved 2D velocity estimation of moving targets and refocusing based on back projection and velocity SAR (another multichannel SAR-GMTI technique). Nevertheless, these methods introduce nonlinear operations, which degrade the performance in the case of low SNR. [18,19,20,21] presented a method for imaging moving targets via the compressive sensing (CS) method, which is capable of generating images with better target focusing, especially with low SNR and high undersampling ratios.

The nonparametric algorithms mainly include the maximum contrast (MC) [22], minimum entropy (ME) [23,24], weighted least-squares (WLS) [25], sharpness optimization [26,27], Doppler centroid tracking (DCT) [28], phase gradient autofocus (PGA) [29] and rank-one phase estimation (ROPE) [30,31] methods. Because the MC, ME, WLS and sharpness optimization methods do not make any assumptions about the characteristics of the target itself, they are highly adaptable. However, since the synthetic aperture process is non-stationary and random, such algorithms generally have local extremum problems. The DCT, PGA, and ROPE methods align the range envelopes and successively adjust the phase to compensate for the translational motion. The DCT and PGA [32] methods are not model-based and exhibit robust performance. Nevertheless, their compensation accuracy is unsatisfactory if there are high-frequency and random phase errors. Furthermore, the model-based ROPE method assumes that each range bin contains no more than one scattering center. The main idea of the method is to use the phase finite difference to estimate the phase error, to find the phase average by alternating along the range direction and the azimuth direction, and to estimate both the phase error and Doppler frequency. In addition, the high azimuth resolution in ISAR processing is generated using a Doppler frequency gradient generated by the rotation of the target relative to the radar line of sight (RLOS) [33]. By averaging all range units of the estimated Doppler frequency, the phase error will be obtained more accurately with influences of the rotational phase weakened, ultimately owning a more precise compensation for the translational phase error. The most remarkable feature of the method is that it estimates not only the phase error of arbitrary order but also the wideband phase error. However, the ROPE method still includes some flaws: The model-based ROPE algorithm strictly requires that there be at most one strong scattering point for each range bin, which limits its application to many images that do not approximate the model; the performance of the method with respect to the phase error estimation will be greatly reduced under low SNR; and the ROPE method uses zero as the initial estimation for the Doppler frequency, which is blind and may lead to unsatisfactory estimates.

Motivated by these aforementioned observations, in this paper, a refocusing method named IRPOE is proposed to solve the above problems existing in ROPE. Our contributions are summarized as follows:

- We use the DCT algorithm in the IROPE method to improve the SNR of the data and render the subsequent estimation more accurate, which also makes the data more consistent with the model of the ROPE method.
- We avoid the averaging of phase vectors with different linear components and maximize the accuracy of the phase compensation by using the circular shift of the prominent point in each range bin to zero frequency to rationalize the initialization of the Doppler center.
- Moreover, better estimates are obtained through iteration. Multiple iterative algorithms improve the SNR, which further improves the accuracy of the Doppler circular shift and the estimation of phase error.
- The proposed method focuses the blurred images well and exhibits the superiority of robustness, reduced sidelobes, and suitability for various phase errors, which does not require time-consuming parameter adjustment procedures to achieve improved performance and allows a long coherent processing interval.

The Gaofen-3 (GF-3) satellite is the first Chinese C-band multi-polarization high-resolution SAR imaging satellite [34]. As one of the most important satellites in China’s Earth observation systems, GF-3’s features include high resolution, large imaging swath, multiple imaging modes, and long operating life [35,36]. GF-3 plays an essential role in the fields of marine environment monitoring, land resource investigation, and disaster prevention, providing high-quality data for scientific experiments. This paper uses the ocean data of the GF-3 satellite to demonstrate the proposed method and the work has the merit to show its potentialities against satellite data.

This paper is organized as follows. The moving signal model in SAR and ISAR systems is presented in Section 2. Section 3 proposes a phase estimation algorithm named IROPE and elaborates the performance. Extensive experimental results on both simulated and real data are presented in Section 4 to demonstrate the effectiveness and robustness of the proposed method. Finally, Section 5 concludes the paper.

## 2. Moving Signal Model

In this section, the signal models of SAR and ISAR are presented. When introducing the SAR signal model, the moving echo signal characteristics and the influence of motion parameters are analyzed. Furthermore, the translation and rotation Doppler shifts involved in the ISAR signal model are analyzed.

### 2.1. SAR Signal Model

#### 2.1.1. Analysis of Moving Echo Characteristics

**Figure 1:**
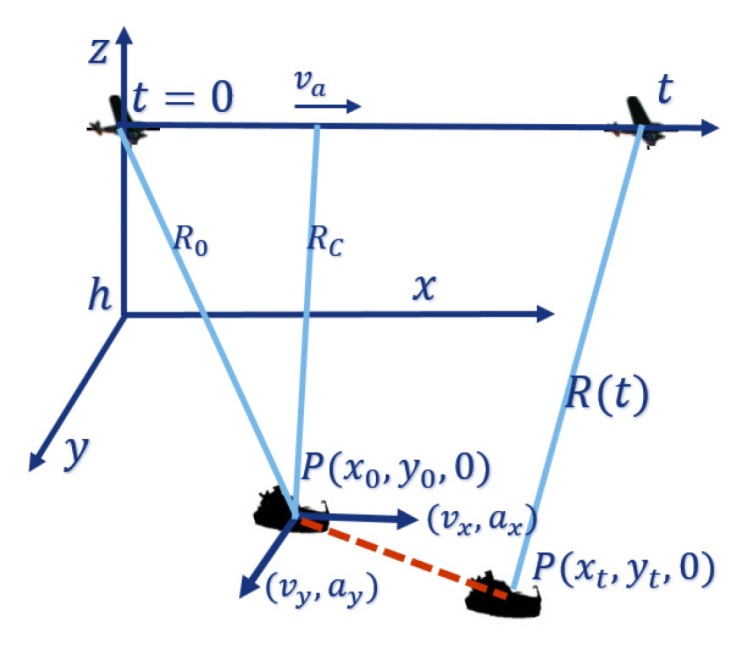
Moving target SAR geometry.

The geometry of SAR imaging of the moving target is shown in Figure 1. The target is described in Cartesian coordinates with the initial position at $P(x_{0},y_{0},0)$. The SAR platform moves along the predetermined track, where $v_{a}$ and *h* represent the velocity and height, respectively. $v_{x}$, $a_{x}$, $v_{y}$, $a_{y}$, $v_{r}$, and $a_{r}$ are the observed target’s velocities and accelerations in azimuth, range and radar RLOS directions. *t* is the slow time, and the distance from point P to the radar platform is $R_{c},R_{c}^{2}=y_{0}^{2}+h^{2}$. $R_{0}$ is the distance between SAR and the target at the initial time, and $R_{0}^{2}=x_{0}^{2}+R_{c}^{2}$. The target moves to $P(x_{t},y_{t},0)$ at time *t*, and the distance between SAR and the target is R(t).

Letting $\hat{v}=v_{a}-v_{x}$, the square of the slant range R(t) is described as

(1) $$\begin{array}{cc} R{\left(t\right)}^{2} & =h^{2}+{\left(v_{a}t-x_{0}-v_{x}t-\frac{1}{2}a_{x}t^{2}\right)}^{2}+{\left(y_{0}+v_{y}t+\frac{1}{2}a_{y}t^{2}\right)}^{2}\\ \; & =R_{0}^{2}+{\left(\hat{v}t-\frac{1}{2}a_{x}t^{2}\right)}^{2}-2x_{0}\left(\hat{v}t-\frac{1}{2}a_{x}t^{2}\right)+{\left(v_{y}t+\frac{1}{2}a_{y}t^{2}\right)}^{2}+2y_{0}\left(v_{y}t+\frac{1}{2}a_{y}t^{2}\right) \end{array}$$

Equation (2) gives Taylor series expansions of R(t) and ignores high-order items (cubic or higher), where $\frac{v_{r}}{v_{y}}=\frac{y_{0}}{R_{0}}$ and $\frac{a_{r}}{a_{y}}=\frac{y_{0}}{R_{0}}$. Equation (1) is simplified as

(2) $$\begin{array}{cc} R(t) & =R_{0}+\frac{1}{2R_{0}}\left(\left({\hat{v}}^{2}+v_{y}^{2}+a_{r}R_{0}+x_{0}a_{x}\right)t^{2}-2x_{0}\hat{v}t\right)+v_{r}t \end{array}$$

Accordingly, the Doppler phase ϕ(t) is

(3) $$\begin{array}{cc} \phi (t) & =\frac{4\pi }{\lambda }R\left(t\right)\\ & =\frac{4\pi }{\lambda }\left(R_{0}+\frac{1}{2R_{0}}\left(\left({\hat{v}}^{2}+v_{y}^{2}+a_{r}R_{0}+x_{0}a_{x}\right)t^{2}-2x_{0}\hat{v}t\right)+v_{r}t\right) \end{array}$$

where λ represents the wavelength of the transmitted signal.

(4) $$\begin{array}{c} \left\{\begin{array}{cc} f_{c} & ={\left.\frac{-1}{2\pi }\frac{d\phi }{dt}\right|}_{t=0}=\frac{-2v_{r}}{\lambda }+\frac{2x_{0}\hat{v}}{\lambda R_{0}}\\ f_{r} & ={\left.\frac{df_{c}}{dt}\right|}_{t=0}=\frac{-2}{\lambda R_{0}}\left({\hat{v}}^{2}+v_{y}^{2}+x_{0}a_{x}+a_{r}R_{0}\right) \end{array}\right. \end{array}$$

where $f_{c}$ represents the Doppler centroid frequency, and $f_{r}$ is the azimuth FM rate.

For stationary targets, $v_{x}=v_{y}=v_{r}=0,a_{x}=a_{y}=a_{r}=0$. Equation (4) is expressed as

(5) $$\begin{array}{c} \left\{\begin{array}{cc} f_{rc} & =\frac{2x_{0}v_{a}}{\lambda R_{0}}\\ f_{rr} & =\frac{-2v_{a}^{2}}{\lambda R_{0}} \end{array}\right. \end{array}$$

Then, the Doppler centroid frequency and the azimuth FM rate generated by the target’s motion are

(6) $$\begin{array}{c} \left\{\begin{array}{cc} f_{lc} & =\frac{-2v_{r}R_{0}-2x_{0}v_{x}}{\lambda R_{0}}\\ f_{lr} & =\frac{-2}{\lambda R_{0}}\left(v_{x}^{2}-2v_{a}v_{x}+v_{y}^{2}+a_{r}R_{0}+x_{0}a_{x}\right) \end{array}\right. \end{array}$$

#### 2.1.2. Analysis of Moving Target Response

Equation (7) gives Taylor series expansions of the phase error:

(7) $$\begin{array}{cc} \Delta \phi (t) & =\frac{4\pi }{\lambda }\Delta R(t)\\ & \approx \frac{4\pi }{\lambda }\left(R+{\left.\frac{d\Delta R(t)}{dt}\right|}_{t=0}t+{\left.\frac{d^{2}\Delta R(t)}{dt^{2}}\right|}_{t=0}\frac{t^{2}}{2}+...\right) \end{array}$$

As [37] mentioned, the first-order phase errors cause azimuth positional offset of the target scattering point, i.e., position deviation. Quadratic phase errors cause target defocusing. Third-order phase errors mainly cause the asymmetry of the sidelobe levels on both sides of the main lobe; in the strong target condition, the image appears ghost-like. The fourth phase errors mainly cause the sidelobe level to increase. The higher-order phase errors will increase the integrated sidelobe level and have little effect on the main lobe width. Generally, due to the Doppler effect, the azimuthal motion of the ship results in blurred defocusing, and the range motion results in an additional shift of the image.

### 2.2. ISAR Signal Model

Assuming that the number of scattering centers is K, the range-compressed data are represented as [37]

(8) $$\begin{array}{c} s\left(\tau ,t\right)=\sum_{k=1}^{K}A_{k}\rho_{r}\left(\tau -2r\left(t\right)/c\right)\omega_{a}\left(t-t_{c}\right)e^{-j\frac{4\pi f_{0}r\left(t\right)}{c}} \end{array}$$

Here, $A_{k}$ represents the backscattered coefficients of the scatterer *k*. $f_{0}$ is the carrier frequency of the system, and τ, *t* and $t_{c}$ denote the fast time, slow time, and beam center offset time, respectively. The distance of point P from the radar is r(t). $\rho_{r}$ represents range envelope (a sinc function) and $\omega_{a}$ represents azimuth envelope (a sinc-squared function) [38].

**Figure 2:**
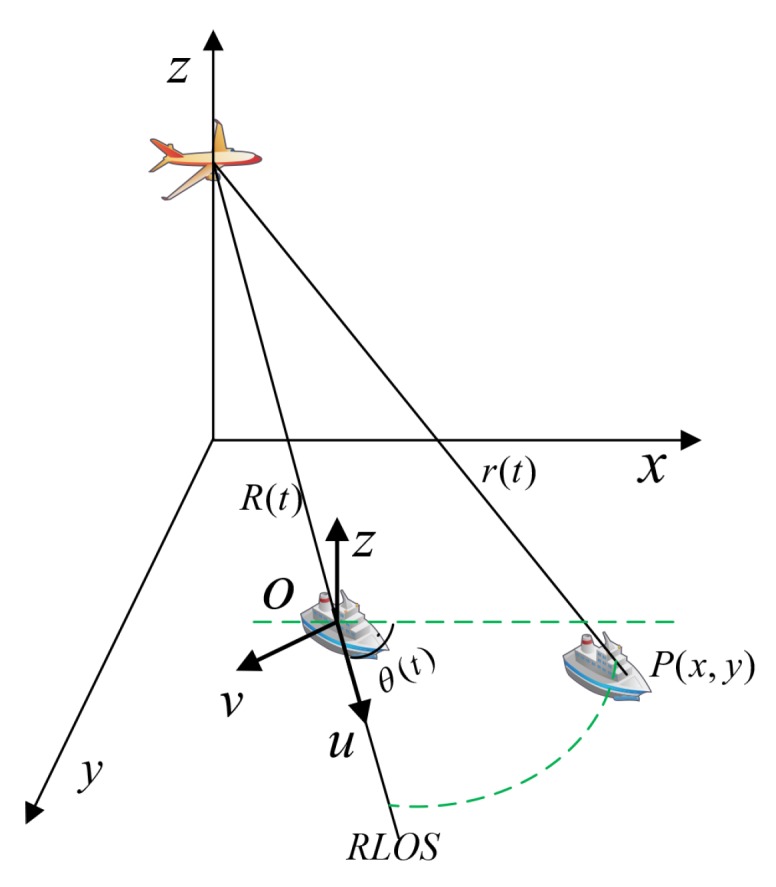
Geometry of the ISAR system.

The Doppler effect of the target’s motion is described in the geometry in Figure 2. From Equation (8), motion compensation removes the phase term $exp(-j4\pi f_{0}r\left(t\right)/c)$. Assuming that the middle of the target is the origin O, r(t) is expressed as

(9) $$\begin{array}{c} r(t)\approx R(t)+xcos\theta (t)+ysin\theta (t) \end{array}$$

Here, R(t) is the target’s translational range distance from the radar and θ(t) represents the rotational angle of the target with respect to the RLOS axis, *u*. Equation (10) gives the Taylor series expansions of R(t) and θ(t) and ignores high-order items (cubic or higher):

(10) $$\begin{array}{c} \begin{array}{c} R\left(t\right)\approx R_{0}+v_{t}t+1/2a_{t}t^{2}+\cdots \\ \theta \left(t\right)\approx \theta_{0}+\omega_{r}t+1/2\alpha_{r}t^{2}+\cdots \end{array} \end{array}$$

$R_{0}$ is the initial range of the target, and $v_{t}$ and $a_{t}$ are the target’s translational velocity and acceleration, respectively. Similarly, $\theta_{0}$ is the initial angle of the target with respect to the RLOS axis. $\omega_{r}$ and $\alpha_{r}$ are the angular velocity and acceleration of the target, respectively.

The echoes of the *k*th range bin are expressed as:

(11) $$\begin{array}{c} s_{k}\left(\tau ,t\right)=A_{k}\rho_{r}\left(\tau -2r\left(t\right)/c\right)\omega_{a}\left(t-t_{c}\right)\phi_{t}\phi_{r} \end{array}$$

where $\phi_{t}$ and $\phi_{r}$ are the phase terms caused by the translational and rotational movement of the target, respectively.

(12) $$\begin{array}{c} \left\{\begin{array}{cc} \phi_{t} & =e^{\frac{-j4\pi f_{0}}{c}\left(R_{0}+v_{t}t+1/2a_{t}t^{2}+\cdots \right)}\\ \phi_{r} & =e^{\frac{-j4\pi f_{0}\sqrt{x^{2}+y^{2}}}{c}sin\left(\beta +\theta_{0}+\omega_{r}t+1/2\alpha_{r}t^{2}+\cdots \right)} \end{array}\right. \end{array}$$

Here, $sin\beta =\frac{x}{\sqrt{x^{2}+y^{2}}}$. The imaging process of the following section removes the influence of the translational phase, including $v_{t}$ and $a_{t}$, which offers no contribution to ISAR imaging [39].

## 3. Improved Rank-One Phase Estimation Algorithm

### 3.1. Problems of the Rank-One Phase Estimation Algorithm

The ROPE method, first developed in [30], estimates and removes the phase error, which guarantees that range-Doppler (RD) imaging can proceed in the usual manner. The algorithm has been modified by [31] to extend its scope of application (including ISAR processing), but some limitations remain. First, the model-based ROPE algorithm strictly requires that there be at most one strong scattering point for each range bin, which limits its application to many images that do not fit the model. Second, the performance of the algorithm decreases sharply under low SNR. Next, blind initialization of the Doppler frequency to zero results in inaccurate estimation. These problems limit the performance and application of the ROPE algorithm. To solve the above problems, the IROPE algorithm is proposed and explained in detail as follows.

### 3.2. Principle of IROPE

Combine formulas to explain the improvements of IROPE and the reasons for the improvement in detail.

**I. Preliminary Phase Compensation**

First, the range-aligned echo signal e(r) is subjected to preliminary phase estimation and compensation using the DCT algorithm.

Multiply the conjugate of the *i*th echo with the next echo to find the average phase difference between adjacent range units:

(13) $$\begin{array}{c} e^{j\varphi }=\frac{\int e_{i}^{*}\left(r\right)e_{i+1}\left(r\right)dr}{\int \left|e_{i}\left(r\right)e_{i+1}\left(r\right)\right|dr} \end{array}$$

Use $e^{j\varphi }$ to adjust the phase shift of $e_{i+1}\left(r\right)$ so that the average phase shift with respect to the adjacent one-dimensional range direction is zero, which is equivalent to aligning the target to a phase center, and the average Doppler shift of the target rotating around the center is zero, thus eliminating the effect of the remaining phase difference.

Through the preliminary phase correction, the SNR is improved, the subsequent estimation becomes more accurate, and the processed image is more consistent with the signal model. After the initial phase correction in this step, the peak value of the special point obtained by Fourier transform will be sharper than the original, which improves the effect of the subsequent circular shifting step.

**II. Two-step Convergence**

Next, there are *J* range units and that each range cell contains no more than one scattering center. The signal of the *j*th range cell is given by

(14) $$\begin{array}{c} s_{k,j}=a_{j}\exp\left[i\left(\omega_{j}k\Delta T+\varepsilon_{k}+\alpha_{j}\right)\right]+n_{k,j} \end{array}$$

where $a_{j}$ is the amplitude of the signal, *k* is the azimuth pulse number, ΔT is the pulse period, $\varepsilon_{k}$ is the phase error, $n_{k,j}$ is the additional complex noise, and $\omega_{j}/2\pi$ is the Doppler position after imaging.

The phase in each pixel of the range-time array is

(15) $$\begin{array}{c} \varphi_{k,j}=\omega_{j}k\Delta T+\varepsilon_{k}+\alpha_{j} \end{array}$$

The difference of Equation (15) is

(16) $$\begin{array}{c} \hat{\varphi_{k,j}}=\varphi_{k+1,j}-\varphi_{k,j}=\omega_{j}\Delta T+\varepsilon_{k+1}-\varepsilon_{k} \end{array}$$

Assume $\hat{\omega_{j}}=\omega_{j}\Delta T$ so that

(17) $$\begin{array}{c} \hat{\varphi_{k,j}}=\varphi_{k+1,j}-\varphi_{k,j}=\hat{\omega_{j}}+\varepsilon_{k+1}-\varepsilon_{k} \end{array}$$

when the SNR is infinite, i.e., $n_{k,j}=0$.

(18) $$\begin{array}{c} D_{k,j}=\frac{s_{k+1,j}s_{k,j}^{*}}{\left|s_{k+1,j}\right|\left|s_{k,j}^{*}\right|}=\exp\left[i\left(\hat{\omega_{j}}+\varepsilon_{k+1}-\varepsilon_{k}\right)\right]=\exp\hat{\varphi_{k,j}}=\exp\left[i\hat{\omega_{j}}\right]\exp\left[i\left(\varepsilon_{k+1}-\varepsilon_{k}\right)\right] \end{array}$$

$D_{k,j}$ is the product of a column vector and a row vector, and $D=\left[D_{k,j}\right]$ is a rank-one matrix.

Let ${\hat{\varepsilon }}_{k}^{\left(p\right)}=\varepsilon_{k+1}-\varepsilon_{k}$. After initialization, the ROPE method consists of the following two operations

(19) $$\begin{array}{c} \left\{\begin{array}{cc} {\hat{\varepsilon }}_{k}^{\left(p\right)} & =∠\sum_{j=1}^{J}D_{k,j}\exp\left(-i2\pi {\hat{\omega_{j}}}^{\left(p-1\right)}\right)\\ {\hat{\omega_{j}}}^{\left(p\right)} & =∠\sum_{k=1}^{K}D_{k,j}\exp\left(-i2\pi {\hat{\varepsilon }}_{k}^{\left(p\right)}\right) \end{array}\right. \end{array}$$

The superscript *p* represents the *p*th operation. When the maximum value of the two estimated changes is less than the small threshold value *T*, the process leads to convergence. When the SNR is high, although the influence of noise causes the rank of matrix D to not be equal to one but approaching one, the two-dimensional alternative estimation is still reasonable and feasible. Nevertheless, when the SNR is low, $n_{k,j}$ cannot be ignored, *D* is not a rank-one matrix, and the ROPE algorithm fails. The final phase error estimate is

(20) $$\begin{array}{c} \hat{\varepsilon }=\sum_{k=1}^{K}{\hat{\varepsilon }}_{k}^{\left(p\right)} \end{array}$$

Equation (19) estimates the phase error and Doppler frequency simultaneously, which estimates the phase error more accurately and avoids the influence of the phase rotation component.

However, the initial phase error ${\hat{E}}_{1}$ in Equation (20) of the original ROPE algorithm is simply set to zero, which is relatively blind and results in unsatisfactory estimation.

**III. Circular Shifting**

The maximum value of the range bin still represents the Doppler frequency corresponding to the strong scattering point, but the energy of the scattering point diffuses in the azimuth direction. Moving the strongest scattering point to zero Doppler frequency eliminates the deficiency of setting the initial Doppler frequency to zero to some extent. The circular shift operation not only aligns the strong scatterers but also improves the SNR of the phase compensation; subsequently, the processed data are more consistent with the model.

**IV. Iteration**

Nevertheless, it is difficult to accurately align the Doppler circular shift when the SNR is low, which affects the estimation accuracy. Therefore, multiple iterative algorithms are then used to improve the SNR, thereby further improving the accuracy of the Doppler circular shift and the estimation of phase error.

The algorithm is summarized in Algorithm 1.
**Algorithm 1:** The IROPE algorithm for phase compensation**Input: The range-aligned echo e(r), [Nr,Na]=size(e(r)), p=0, number of iterations *l*, threshold value *T*****1: I. Preliminary Phase Compensation****2: for i=1:Na−1****3: $e_{i+1}\left(r\right)=e_{i+1}\left(r\right).*e^{j\varphi }$****4: end****5: IV. Iteration****6: for l=1:l (Image entropy is further applied to control the iteration process)****7:  II. Two-step Convergence****8:  III. Circular Shifting****9:  while ${\hat{\varepsilon }}_{k}^{\left(p\right)}-{\hat{\varepsilon }}_{k}^{\left(p-1\right)}$> T****10:   for k=1:Na−1****11:    Update ${\hat{\varepsilon }}_{k}^{\left(p\right)}$** calculated by Equation (19)**12:   end****13:   for $j=1:N_{r}$****14:    Update $\omega_{j}^{\left(p\right)}$** calculated by Equation (19)**15:   end****16:   p=p+1****17:  end while****18:  $\hat{\varepsilon }=\sum_{k=1}^{K}{\hat{\varepsilon }}_{k}^{\left(p\right)}$****19:  $e\left(r\right)=e\left(r\right).*exp\left(-1i*\hat{\varepsilon }\right)$****20: end****Output: Compensated range-Doppler echo e(r), phase error $\hat{\varepsilon }$**

**Figure 3:**
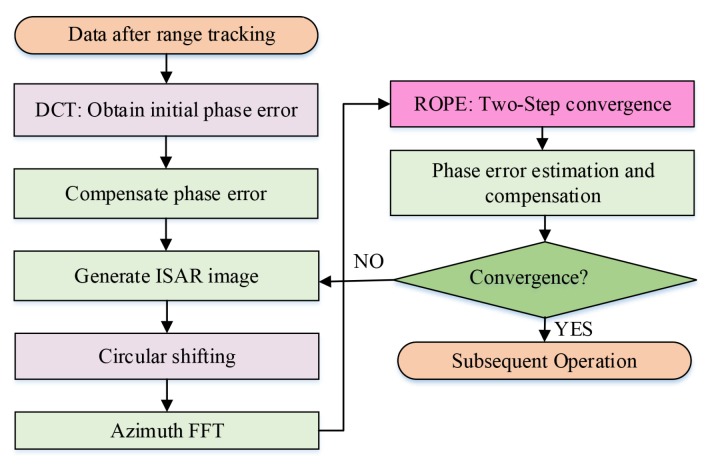
Block diagram of the IROPE procedure.

The flow chart of the IROPE procedure is indicated in Figure 3 and described in detail as follows.

Step 1: Use the DCT method to perform initial phase correction on the echo data after range tracking for preprocessing.

Step 2: Perform IFFT transform in the azimuth direction to generate an ISAR image.

Step 3: Find the maximum amplitude and set the initial zero Doppler to the circular shift of the prominent point in each range bin.

Step 4: By performing azimuth FFT, the data are transformed to the range-Doppler domain.

Step 5: Use two-step convergence approach to obtain and compensate for the phase error.

Step6: If the effect of refocusing is not sufficient, repeat the process from Step2 to Step5.

### 3.3. Performance of IROPE

This subsection presents the experimental results based on range-aligned echo to illustrate the performance of IROPE. The radar operates in the X band. The transmitted signal bandwidth and the synthetic aperture time are 100 MHz and 3.32 s, respectively. The translational motion of the target with range velocity of $v_{y}$ = 6 m/s, azimuth velocity of $v_{x}$ = 15 m/s, and azimuth acceleration of $a_{x}$ = 2 m/s${}^{2}$ is determined. Assume that there is a sinusoidal error term caused by the target’s rotation, which is chosen as $\frac{0.5\pi }{180}sin\left(0.6t\right)$ rad. The rotational velocity is 0.6 rad/s and *t* represents the azimuth observation time. The pulse repetition frequency (PRF) and the radar velocity are 600 Hz and 250 m/s, respectively.

**Figure 4:**
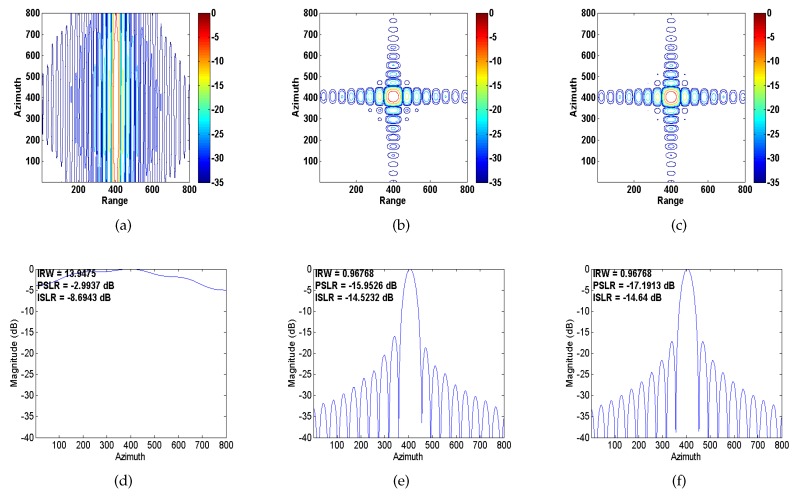
The interpolated contour and azimuth profile. (**a**,**d**) Conventional SAR processing; (**b**,**e**) ROPE; (**c**,**f**) IROPE.

*Example 1*Figure 4 shows the experimental results corresponding to a single moving point target. It can be seen that the point is well focused by the ROPE and IROPE methods. Comparing the interpolated contour and the value of PSLR, IROPE exhibits slight superiority over ROPE. (The technical indicators shown in the figure are explained as follows: impulse response width (IRW), namely the 3 dB main lobe width of impulse response; peak sidelobe ratio (PSLR), the height ratio of the maximum sidelobe to the main lobe; and integrated sidelobe ratio (ISLR)).

**Figure 5:**
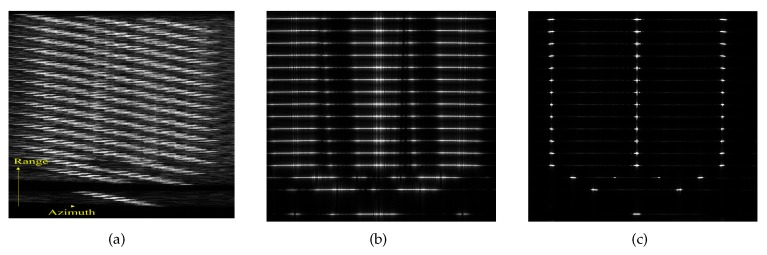
Refocused performance. (**a**) Conventional SAR processing; (**b**) ROPE; (**c**) IROPE.

*Example 2*Figure 5 shows the experimental results for a simulated ship, i.e., multiple-point targets, in which each range bin of the ship’s hull has three strong scattering points with the same strength. It can be seen from Figure 5b that the ROPE algorithm fails because of not satisfying the model in which each range cell contains no more than one scattering center. According to the above subsection analysis, IROPE compensates for the deficiency of ROPE and obtains a good focusing effect.

**Figure 6:**
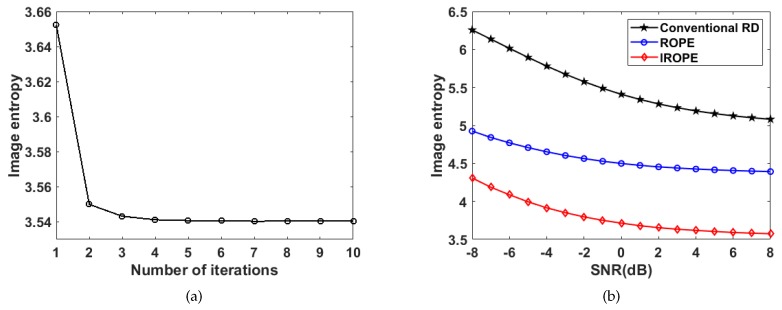
Variation in image entropy in terms of iterations (**a**) and SNRs (**b**).

Figure 6a shows that the image entropy (defined in Equation (21)) decreases with the increase of IROPE’s iteration times, which proves that iteration improves the image focusing effect. Figure 6b exhibits the image entropy processed by different algorithms, which changes with SNR. The results prove the robust performance with respect to the noise of the IROPE algorithm.

The phase error estimation performance is provided in the next section.

### 3.4. The Whole Process of the Refocusing Method

**Figure 7:**
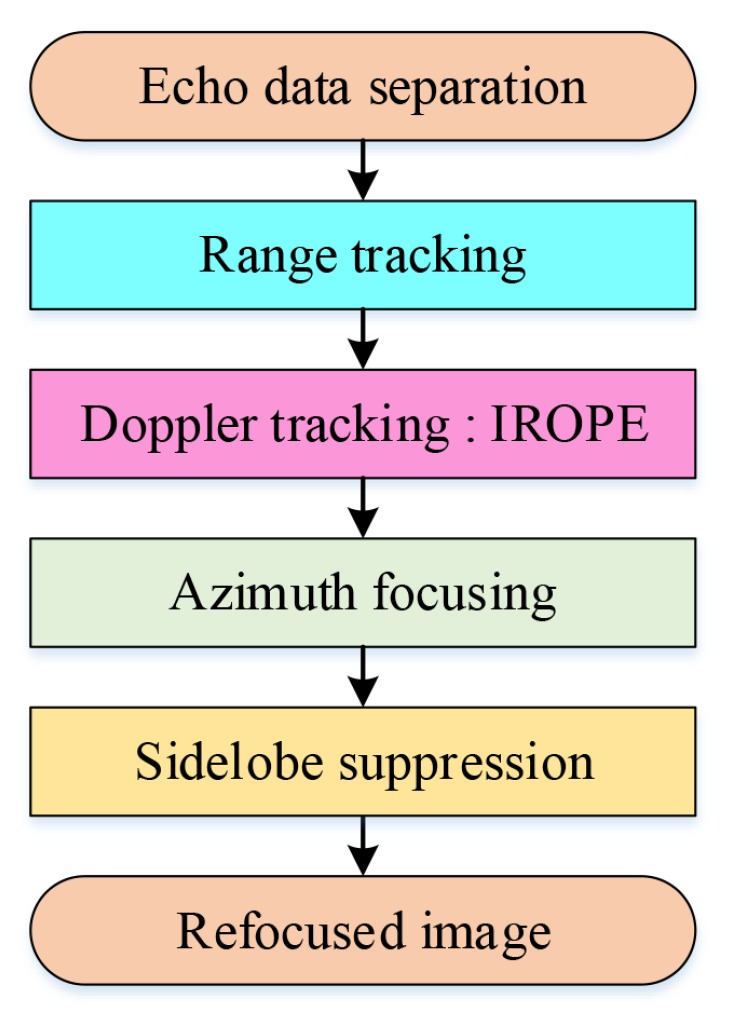
Block scheme of the whole process of the refocusing method.

The whole imaging flow chart is shown in Figure 7.

First, the separated echo signal is obtained. The echo data are obtained from the original raw echo data or complex image data. The former approach implements range compression on the original raw echo data, while the latter performs azimuth inverse compression on the selected complex image data of the target of interest to acquire the required echo data for subsequent operations.

Second, range tracking is performed on echo data. Using the cross-correlation [40] of the average range profile, a real-time and efficient method, the ship echo is correlated with the first echo in the imaging time. In addition, through range alignment, the range units of the echoes are aligned, and the amplitude and phase changes of the echo range sequence of each range unit are normal. Eventually, the phase change process generated by the target translation is retained.

Furthermore, we apply IROPE to estimate phase error ${\hat{\varepsilon }}_{k}$ and obtain the compensated range-Doppler echo e(r).

Next, we combine the conventional RD algorithm and use the Hamming window to achieve well-focused images and suppress sidelobes. The conventional RD algorithm is applied to obtain focused ISAR images if the maritime target moves smoothly. However, if the target maneuvers or undergoes significant angular motions (roll, pitch, and yaw), the RD technique does not function properly, and the time-frequency analysis [41] method is a better choice.

## 4. Experiments and Performance Comparisons

In this section, the robustness and effectiveness of the proposed method are verified by simulation experiments. Then, the results based on the spaceborne SAR data acquired by the GF-3 SAR system are demonstrated.

### 4.1. Results of Spotlight Simulation

The proposed method is applied to spotlight simulation data and analyzed for different motions of the ship target by comparing it with other refocusing algorithms. The basic parameters of the spotlight simulation are shown in Table 1.

#### 4.1.1. Ship Target with Velocity and Acceleration

**Figure 8:**
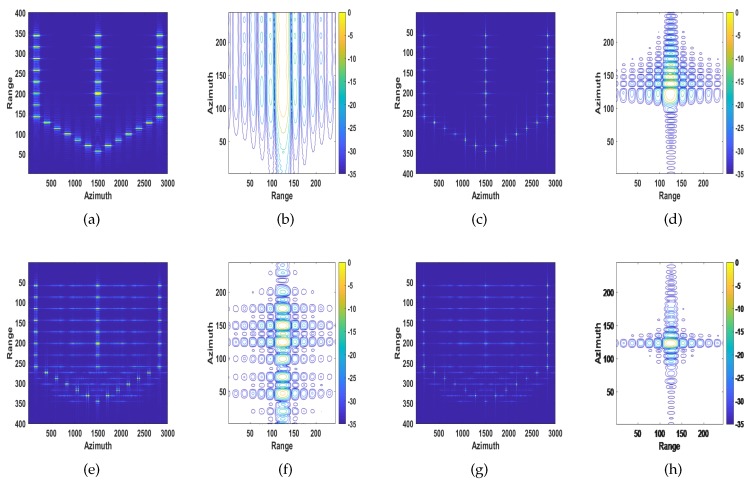
Recovered images of defocused ship target with velocity and acceleration. (**a,b**) Conventional SAR processing system; (**c,d**) MD method; (**e,f**) ROPE method; (**g,h**) The proposed method.

The target is moving away from the radar with range velocity of $v_{y}$ = 3 m/s, azimuth velocity of $v_{x}$ = 15 m/s and azimuth acceleration of $a_{x}=2$ m/s${}^{2}$. As mentioned in Section 1 and Section 2, the quadratic phase errors caused by velocity lead to image defocusing; cubic phase error introduced by acceleration mainly causes the asymmetry of the sidelobe levels on both sides of the main lobe [37]. The conventional SAR image and the recovered images of MD, ROPE and the proposed algorithm are shown in Figure 8. It can be seen from Figure 8c,d that the MD algorithm only compensates for the quadratic phase errors caused by velocity but cannot compensate for the cubic phase error caused by acceleration. The image processed by the ROPE algorithm has a high energy of the sidelobes, as shown in Figure 8e,f. Based on the above analysis, the image quality of the proposed method is superior to the other methods.

**Figure 9:**
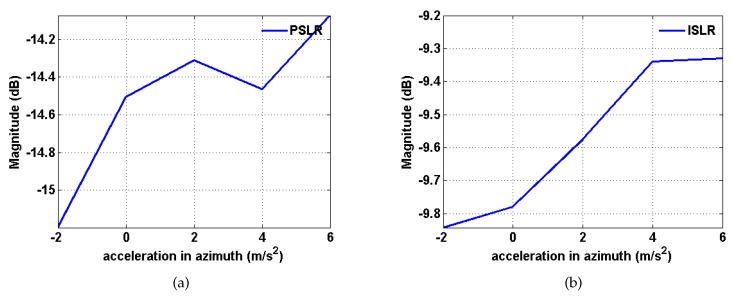
Variations in the magnitudes of PSLR and ISLR in terms of the linear acceleration based on the proposed method. (**a**) PSLR; (**b**) ISLR.

A discussion of what amount of non-uniform motion can be effectively analyzed during the long CPI, i.e., the limitation in azimuth linear acceleration, follows. Azimuth linear acceleration varies from −2 to 6 m/s${}^{2}$ according to a step size of 2 m/s${}^{2}$ [42]. The variations in the magnitudes of PSLR and ISLR are shown in Figure 9. It can be seen that the magnitudes of PSLR and ISLR fluctuate with increasing acceleration, and the overall trend is upward. The maximum magnitude of PSLR is below −14 dB, while the maximum magnitude of ISLR is below −9 dB, which means that the algorithm is suitable for practical situations.

#### 4.1.2. Ship target with Translation and Rotation

**Figure 10:**
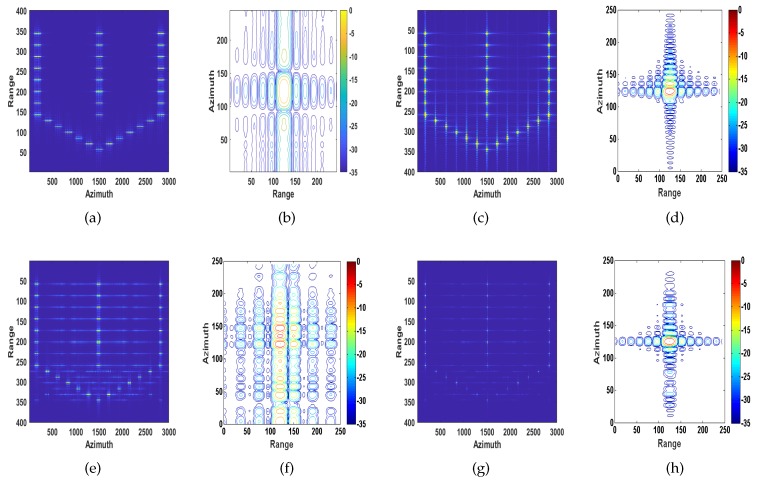
Recovered images of defocused ship target with translation and rotation speed. (**a**,**b**) Conventional SAR processing system; (**c**,**d**) MD method; (**e**,**f**) ROPE method; (**g**,**h**) The proposed method.

The target is moving away from the radar with range velocity of $v_{y}$ = 3 m/s and azimuth velocity of $v_{x}$ = 15 m/s. We assume that there is a sinusoidal error term caused by the target’s rotation, which is chosen as $\frac{0.5\pi }{180}sin\left(0.6t\right)$ rad. High-frequency and wideband phase errors introduced by rotational velocity mainly affect the sidelobe region and increase the sidelobe level [37]. The conventional SAR image and the recovered images of MD, ROPE and the proposed algorithm are shown in Figure 10. It can be seen from Figure 10c-d that the MD algorithm only compensates for the quadratic phase errors caused by velocity and cannot compensate for the high-frequency and wideband phase errors introduced by rotational velocity. From Figure 10e-f, processed by the ROPE method, the main lobe is almost submerged by the sidelobes. Comparing the values of PSLR and ISLR for different algorithms, the image quality of the proposed algorithm is also superior to the other approaches.

**Figure 11:**
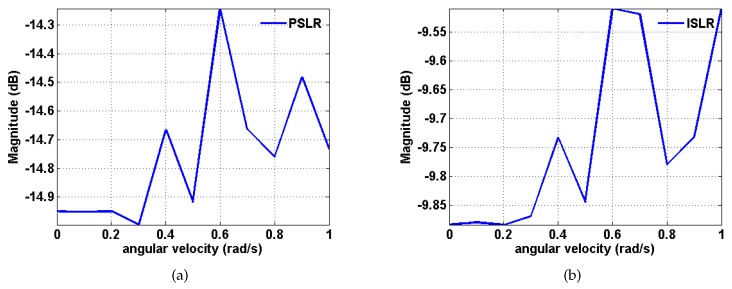
Variation in the magnitudes of PSLR and ISLR in terms of the rotational angular velocity based on the proposed method. (**a**) PSLR; (**b**) ISLR.

Moreover, assume that $\omega_{r}=\frac{0.5\pi }{180}sin\left(wt\right)$ rad. *w* varies according to a step size of 0.1 rad/s from 0 to 1 rad/s [43]. The experimental results are shown in Figure 11. It can be seen that the magnitudes of PSLR and ISLR fluctuate with increasing rotational angular velocity, and the overall trend is upward. It is observed that the magnitudes of PSLR and ISLR fluctuate with increasing acceleration, and the overall trend is upward. The maximum magnitude of PSLR is below −14 dB, while the maximum magnitude of ISLR is below −9.5 dB, which means that the algorithm is suitable for practical situations.

### 4.2. Spaceborne SAR Data Experiments

In this subsection, the results based on the spaceborne SAR data acquired by the GF-3 SAR system are demonstrated. The parameters are shown in Table 2. The synthetic aperture time has a relatively large value of 8.58 s. In addition to visual inspection, image quality is also evaluated by entropy, contrast and the peak value of the intensity image [44], which are referred to as image quality evaluation metrics (IQEMs).

Let I(m,n) be the absolute value of a two-dimensional complex image, where *m* is the range sample number and *n* is the azimuth number. The image entropy (IE) [23] is written as follows:

(21) $$IE\left(I\right)=-\sum_{m=1}^{M}\sum_{n=1}^{N}\frac{{\left|I\left(m,n\right)\right|}^{2}}{\alpha_{I}}\ln\frac{{\left|I\left(m,n\right)\right|}^{2}}{\alpha_{I}}$$

where $\alpha_{I}$ is the total energy of the image, explained as follows:

(22) $$\alpha_{I}=\sum_{m=1}^{M}\sum_{n=1}^{N}{\left|I\left(m,n\right)\right|}^{2}$$

When the image is well focused, the entropy value is small because of the uniform distribution.

The image contrast (IC) is defined as follows [45]:

(23) $$IC\left(I\right)=\frac{\sqrt{E\{{\left[I\left(m,n\right)-E\left\{I\left(m,n\right)\right\}\right]}^{2}\}}}{E\{I(m,n)\}}$$

when the image is focused correctly, it comprises several significant peaks, which enhances the contrast.

The peak value of the intensity image (IP) is an indicator of the image focusing of a local area of the image, and the calculation expression is

(24) IP(I)=10·log10(max(I(m,n)))

The larger the IP is, the better the image focus is.

Hence, to present a more intuitive and quantitative comparison, Table 3 and Table 4 provide the difference of IQEMs between the processed and the original images—contrast increase, entropy reduction, and IP increase. The higher the value is, the better the image quality is.

#### 4.2.1. Real Data Corrupted by Phase Error

**Figure 12:**
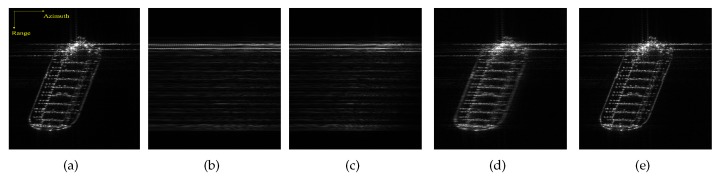
Nominal, corrupted, and recovered images. (**a**) Nominal; (**b**) Corrupted by phase error; (**c**) Image recovered with MD method; (**d**) Image recovered with ROPE method; (**e**) Image recovered with the proposed method.

**Figure 13:**
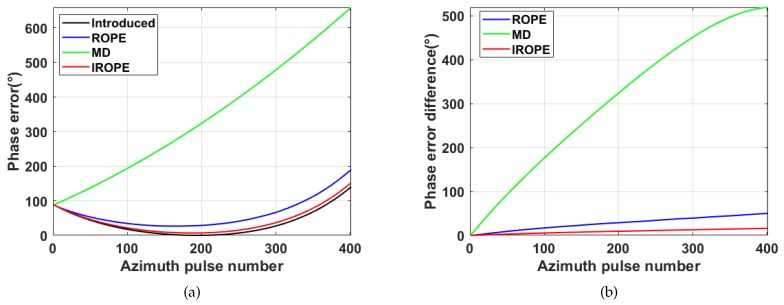
Phase error curve. (**a**) Introduced and estimated phase error; (**b**) Phase error difference.

Figure 12 and Figure 13 show the experimental image results for phase error and demonstrate the capability to estimate the phase error of the proposed method. The phase errors of the quadratic, third and fourth superpositions of the same weight are added to Figure 12a. Figure 12b shows the corrupted image. There is no improvement in the image after the MD method refocusing, as can be seen from Figure 12c. The ROPE method exhibits a better focusing effect than the MD method does, but it is visually worse than the proposed method. A comparison of the image recovered using the proposed method with the original image shows very good agreement, although there are some slight differences. Figure 13 demonstrates that the phase error estimated by IROPE is closest to the introduced phase error. MD only compensates for the quadratic phase errors, so the estimated and true phase errors display considerable deviation. The difference phase error also exhibits a large deviation of ROPE because the actual image does not satisfy the model. The success of the IROPE algorithm is obvious; a bias exists, but the bias does not affect the quality of the recovered image from Table 3.

#### 4.2.2. Intrinsically Corrupted Real Data

**Figure 14:**
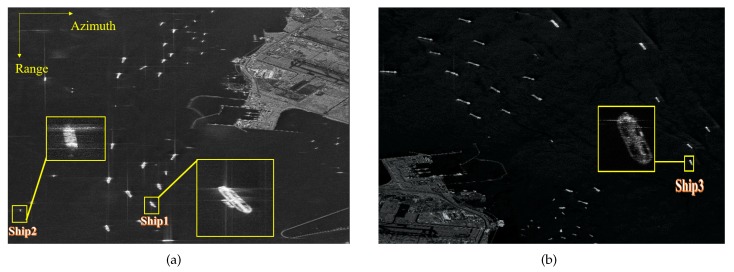
GF-3 SAR image of the Port of Singapore. The yellow rectangles are the enlarged defocused sub-images. (**a**) (E104.0, N1.3); (**b**) (E104.1, N1.3).

Figure 14 illustrates C-band, spotlight GF3 SAR images with azimuth resolution of 1 m.

The center latitude and longitude of the photographed area are (E104.0, N1.3) and (E104.1, N1.3), respectively, located at the Port of Singapore near Changi Airport. This location is at the southern end of the Malay Peninsula, the entrance to the Straits of Malacca.

Three representative defocused ships, marked as Ship1, Ship2 and Ship3, are selected from Figure 14 for the experiment.

**Figure 15:**
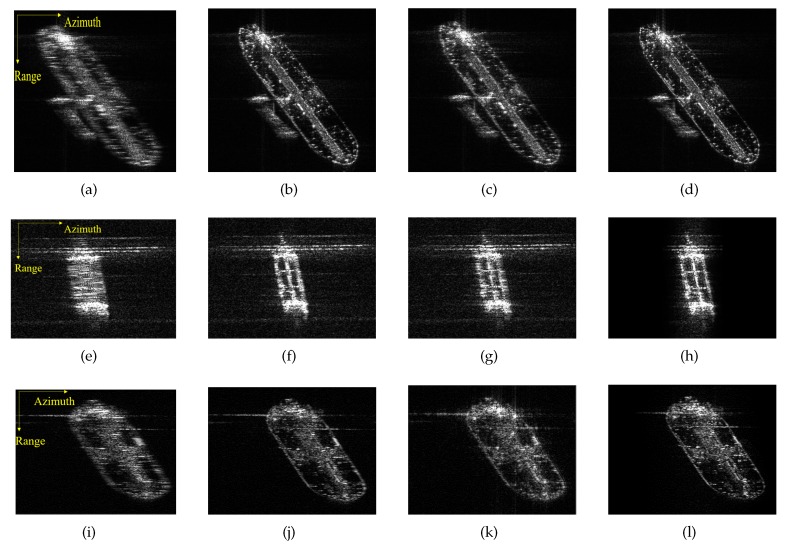
Original and refocused images. (**a**–**d**) Ship1; (**e**–**h**) Ship2; (**i**–**l**) Ship3.

Figure 15 gives the original and refocused images, in which the first column shows the original images and the second, third, and fourth columns present the images obtained by MD, ROPE, and IROPE, respectively. From the defocused images of Ship1 and Ship2, the outlines of these large petroleum tankers are vague, and the details are unrecognizable. The wake of Ship3 is obvious due to the smooth sea conditions and the rapid speed. It is determined that the visual quality of every original image after refocusing is improved; the details and contours of the targets are apparent, which is more conducive to subsequent use. Nevertheless, the images processed by the ROPE method are not well focused and remain slightly blurry. A detailed look reveals that the edge of every ship is processed worse by MD than by the proposed method. The values of the quality metrics in Table 4 indicate that the ship reconstructed by the proposed method, is better focused than that reconstructed by the other methods. The entropy, contrast, and IP are superior in our case.

The experimental results for real data demonstrate the effectiveness and superiority of the IROPE algorithm. The three ships used in the experiment are characterized by various phase errors, with multiple strong scattering points in each range bin under low SNR condition. In these cases, the MD and ROPE algorithms fail, and the proposed method exhibits a better refocusing effect.

## 5. Discussion

In this paper, we studied the topic of how to refocus and accurately image a moving ship which cannot be focused by using conventional SAR. The proposed method is compared with the MD [10,11] method and the ROPE [30,31] method and the comparison result is shown in Table 5: Experimental results show that the performance of MD and ROPE in phase error estimation and accuracy are unsatisfactory. The sub-aperture correlation operation of the MD method only compensates for quadratic phase errors, and real SAR images do not fit well with the narrowly defined ROPE model, which limit their applications.

In view of above-mentioned problems, we have made improvements in preprocessing, circular shifting and iteration based on two-step convergence, which is embodied in the following aspects: improving the SNR and the accuracy of estimation through the DCT method for preliminary phase compensation; eliminating the shortcoming of setting the initial zero Doppler through the center shift of the strongest scattering point of each range bin and enhancing the performance of the method through several iterations. With these improvements, our proposed IROPE can achieve more complete image recovery for SAR images. The application scope and further development of the ROPE method are promoted through our improvement. Meanwhile, the experiment on adding phase error to the real data shows that IROPE estimates the phase error and compensates for arbitrary phase errors more accurately.

Moreover, many studies have been taken on this topic [15,16,19,22,24]. Nevertheless, these methods are based on low-resolution, short-CPI simulation or airborne SAR data, and their application to high-resolution, long-CPI spaceborne SAR images requires further verification. However, the proposed method processes high-resolution spaceborne SAR images, which is verified with GF-3 satellite data. Next, our results suggest a possibility of applying to the spaceborne SAR system with a long CPI (8.58 *s*). Furthermore, our work demonstrates great potency of the application of the high-resolution (1 *m*) SAR images.

However, the proposed method also has shortcomings. On the one hand, the dimensional classes of the ships in the experiments need further confirmation. On the other hand, although the proposed method allows a long coherent processing interval and performs well for maritime targets in stable sea conditions, it still needs further research and improvement for maneuvering targets that are experiencing high sea conditions. Therefore, in the future, we will focus our efforts on solving these problems like engaging the verification of the stated ship type in the AIS signals [27] and the optical photographs [46], and exploring the algorithm of maneuvering targets.

## 6. Conclusions

During the detection of marine moving targets, both SAR and targets are in motion, and thus, conventional SAR processing that only images stationary targets achieves unsatisfactory performance. In this paper, we combined SAR and ISAR techniques and proposed a hybrid SAR/ISAR approach, as the core of the IROPE, to refocus the maritime moving targets. The proposed IROPE method overcomes the weakness of the original ROPE method. Experiments based on simulation and GF-3 measured data demonstrate the effectiveness of the proposed method. In summary, our work makes contributions to the improvement of the unsatisfactory method and the proposed method is also suitable for high-resolution long-CPI spaceborne radar.

## Figures and Tables

**Table 1:** Parameters of spotlight simulation.

Parameter	Value
Mode	Spotlight
Radar Center Frequency (GHz)	5.4
Wavelength (m)	0.0555
Bandwidth (MHz)	50
Azimuth Resolution (m)	1.5
Range Resolution (m)	2.6562
PRF (Hz)	3125
Upsampled PRF (Hz)	9950.2398
Upsampled Doppler Bandwidth (Hz)	6188.7356
Slant-Range (m)	1,067,731.2395
Synthetic Aperture Time (s)	2.3342
SAR Velocity (m/s)	7500
squint angle ()	0

**Table 2:** Parameters of the GF-3 SAR System.

Parameter	Value
Mode	Spotlight
Radar Center Frequency (GHz)	5.400012
Bandwidth (MHz)	240.000000
Azimuth Resolution (m)	1
Range Resolution (m)	0.6
PRF (Hz)	3125.164062
Synthetic Aperture Time (s)	8.58
Satellite Velocity (m/s)	7570.962970

**Table 3:** IQEMs of the corrupted and recovered images.

IQEMs	Corrupted image	MD	ROPE	IROPE
Contrast increase	−4.08	−3.86	−0.47	−0.09
Entropy reduction	−1.69	−1.58	−0.21	0.08
IP increase	−20.01	−16.82	−2.58	0.77

**Table 4:** IQEMs of the GF-3 ship.

Ship	Ship1			Ship2			Ship3		
IQEMs	MD	ROPE	IROPE	MD	ROPE	IROPE	MD	ROPE	IROPE
Contrast increase	0.42	0.23	0.49	0.01	0.01	0.02	0.23	−0.12	0.93
Entropy reduction	0.40	0.25	0.47	0.16	0.15	0.24	0.20	−0.02	0.47
IP increase	6.08	5.21	6.78	3.05	2.60	4.82	4.18	1.53	4.69

**Table 5:** Comparison with other refocusing algorithms.

Works	Schemes	Application	Phase Error Estimation	*Accuracy*
MD	Sub-aperture correlation	Limited	Unsatisfactory	Unsatisfactory
ROPE	Two-step convergence	Limited	Unsatisfactory	Unsatisfactory
IROPE	Preprocessing+ Circular Shifting +Two-step convergence+Iteration	Wide	Good	Good

## References

[1] Moreira A., Prats-Iraola P., Younis M., Krieger G., Papathanassiou K.P. (2013). A Tutorial on Synthetic Aperture Radar. IEEE Geosc. Rem. Sen. M..

[2] Rong J., Wang Y., Han T. (2019). Iterative Optimization-Based ISAR Imaging With Sparse Aperture and Its Application in Interferometric ISAR Imaging. IEEE Sens. J..

[3] Noviello C., Fornaro G., Martorella M., Reale D. IS. Proceedings of the Geoscience and Remote Sens. Symposium (IGARSS).

[4] Jiang Y., Wang H. (2017). Hybrid SAR/ISAR imaging of ship targets based on parameter estimation. Remote Sens. Lett..

[5] Martorella M., Giusti E., Berizzi F., Bacci A., Mese E.D. (2012). ISAR based techniques for refocusing non-cooperative targets in SAR images. IET Radar Sonar Navig..

[6] Chen V.C., Liu B. Hybrid SAR/ISAR for distributed ISAR imaging of moving targets. Proceedings of the 2015 IEEE Radar Conference (RadarCon).

[7] Sun H., Zhou L., Ma J., Liu X. The hybrid SAR-ISAR imaging algorithm applied to SAR moving target imaging. Proceedings of the 2012 9th International Conference on Fuzzy Systems and Knowledge Discovery.

[8] Kim J., Hwang I., Jo H., Kim G., Yoo J.S., Yu J. Phase error compensation in fourier domain for fast autofocus of spotlight SAR. Proceedings of the 2017 USNC-URSI Radio Science Meeting (Joint with AP-S Symposium).

[9] Brown W.M., Palermo C.J. (2007). Effects of Phase Errors on Resolution. IEEE Trans. Mil. Electron..

[10] Calloway T.M., Donohoe G.W. (1994). Subaperture autofocus for synthetic aperture radar. IEEE Trans. Aerosp. Electron. Syst..

[11] Samczynski P., Kulpa K. The use of the Coherent MapDrift technique for SAR image focusing on the sea surface. Proceedings of the 2010 IEEE Radar Conference.

[12] Koo V.C., Chan Y.K., Chuah H.T. (2006). Multiple Phase Difference Method for Real-Time SAR Autofocus. J. Electromagn. Waves Appl..

[13] Pan Z., Fan H., Zhang Z. (2020). Nonuniformly-Rotating Ship Refocusing in SAR Imagery Based on the Bilinear Extended Fractional Fourier Transform. Sensors.

[14] Chen Y., Li G., Zhang Q., Sun J. (2017). Refocusing of Moving Targets in SAR Images via Parametric Sparse Representation. Remote Sens..

[15] Wang Y., Jiang Y. (2011). Inverse Synthetic Aperture Radar Imaging of Maneuvering Target Based on the Product Generalized Cubic Phase Function. IEEE Geosci. Remote Sens. Lett..

[16] Wan J., Zhou Y., Zhang L., Chen Z., Yu H. (2019). Efficient Algorithm for SAR Refocusing of Ground Fast-Maneuvering Targets. Remote Sens..

[17] Tang X., Zhang X., Shi J., Wei S., Tian B. (2019). Ground Moving Target 2-D Velocity Estimation and Refocusing for Multichannel Maneuvering SAR with Fixed Acceleration. Sensors.

[18] Giusti E., Wei Q., Bacci A., Tomei S., Martorella M. Super resolution ISAR imaging via Compressing Sensing. Proceedings of the EUSAR 2014; 10th European Conference on Synthetic Aperture Radar.

[19] Zhao L., Wang L., Bi G., Yang L. (2014). An Autofocus Technique for High-Resolution Inverse Synthetic Aperture Radar Imagery. IEEE Trans. Geosci. Remote Sens..

[20] Liu Y., Quan Y., Li J., Zhang L., Xing M. SAR imaging of multiple ships based on compressed sensing. Proceedings of the 2009 2nd Asian-Pacific Conference on Synthetic Aperture Radar.

[21] Zhang Y., Xing M., Ye P. Joint Rotation Parameters Estimation and High Resolution Imaging for Maneuvering Targets. Proceedings of the EUSAR 2018, 12th European Conference on Synthetic Aperture Radar.

[22] Jing Y., Li Y. Moving target parameter estimation algorithm using contrast optimization. Proceedings of the International Bhurban Conference on Applied Sciences and Technology.

[23] Xi L., Guosui L., Ni J. (1999). Autofocusing of ISAR images based on entropy minimization. IEEE Trans. Aerosp. Electron. Syst..

[24] Huang X., Ji K., Leng X., Dong G., Xing X. (2019). Refocusing Moving Ship Targets in SAR Images Based on Fast Minimum Entropy Phase Compensation. Sensors.

[25] Hui Y., Wenying W., Long Z., Wanming L., Xin N., Pin L. (2019). Improved weighted least-squares phase unwrapping method for interferometric SAR processing. J. Eng..

[26] Morrison R.L., Do M.N., Munson D.C. (2007). SAR image autofocus by sharpness optimization: A theoretical study. IEEE Trans. Image Process..

[27] Sommer A., Ostermann J. (2019). Backprojection Subimage Autofocus of Moving Ships for Synthetic Aperture Radar. IEEE Trans. Geosci. Remote Sens..

[28] Ye C.M., Jia X., Peng Y.N., Wang X.T. Improved Doppler centroid tracking for ISAR based on target extraction. Proceedings of the 2008 IEEE Radar Conference.

[29] Wahl D.E., Eichel P., Ghiglia D., Jakowatz C. (1994). Phase gradient autofocus-a robust tool for high resolution SAR phase correction. IEEE Trans. Aerosp. Electron. Syst..

[30] Dyson F., Garwin R., Horowitz P., Katz J., Muller R., Press W., Weinberger P. (1992). Recovery of SAR Images with One Dimension of Unknown Phases.

[31] Snarski C.A. (1996). Rank one phase error estimation for range-Doppler imaging. IEEE Trans. Aerosp. Electron. Syst..

[32] Ji-Ming Q., Xu H.Y., Liang X.D., Li Y.L. (2015). An Improved Phase Gradient Autofocus Algorithm Used in Real-time Processing. J. Radars.

[33] Gao Y., Xing M., Zhang Z., Guo L. (2019). ISAR Imaging and Cross-Range Scaling for Maneuvering Targets by Using the NCS-NLS Algorithm. IEEE Sens. J..

[34] Han B., Ding C., Zhong L., Liu J., Qiu X., Hu Y., Lei B. (2018). The GF-3 SAR Data Processor. Sensors.

[35] Sun J., Yu W., Deng Y. (2017). The SAR Payload Design and Performance for the GF-3 Mission. Sensors.

[36] Zheng M., Yan H., Zhang L., Yu W., Deng Y., Wang R. (2018). Research on Strong Clutter Suppression for Gaofen-3 Dual-Channel SAR/GMTI. Sensors.

[37] Quegan S., Carrara W.G., Goodman R.S., Majewski R.M. (1995). Spotlight Synthetic Aperture Radar: Signal Processing Algorithms.

[38] Cumming I.G., Wong F.H. Digital Processing of Synthetic Aperture Radar Data: Algorithms and Implementation. https://pdfs.semanticscholar.org/23eb/33af4f0495edff01402ec8eb019e80717897.pdf.

[39] Zheng J., Liu H., Liu Z., Liu Q.H. (2017). ISAR Imaging of Ship Targets Based on an Integrated Cubic Phase Bilinear Autocorrelation Function. Sensors.

[40] Xing M.D., Zheng B., Ming Z.Y. (2001). Range Alignment Using Global Optimization Criterion in ISAR Imaging. Acta Electron. Sin..

[41] Vehmas R., Jylha J., Vaila M., Vihonen J., Visa A. (2018). Data-Driven Motion Compensation Techniques for Noncooperative ISAR Imaging. IEEE Trans. Aerosp. Electron. Syst..

[42] Rong J., Wang Y., Han T. (2020). Interferometric ISAR Imaging of Maneuvering Targets With Arbitrary Three-Antenna Configuration. IEEE Trans. Geosci. Remote Sens..

[43] Zheng J., Su T., Zhang L., Zhu W., Liu Q.H. (2014). ISAR Imaging of Targets With Complex Motion Based on the Chirp Rate Quadratic Chirp Rate Distribution. IEEE Trans. Geosci. Remote Sens..

[44] Martorella M., Berizzi F., Haywood B. (2005). Contrast maximization based technique for 2-D ISAR autofocusing. IEE P-Radar Son. Nav..

[45] Wu W., Hu P., Xu S., Chen Z., Chen J. (2017). Image registration for InISAR based on joint translational motion compensation. IET Radar Sonar Navig..

[46] Hajduch G., Le Caillec J., Garello R. (2004). Airborne high-resolution ISAR imaging of ship targets at sea. IEEE Trans. Aerosp. Electron. Syst..

